# Tafamidis in octogenarians with wild-type transthyretin cardiac amyloidosis: an international cohort study

**DOI:** 10.1093/eurheartj/ehae923

**Published:** 2025-02-26

**Authors:** Philippe Debonnaire, Karl Dujardin, Nicolas Verheyen, Anne-Catherine Pouleur, Steven Droogmans, Mathias Claeys, Alexandre Bohyn, Kris Bogaerts, Milad El Haddad, Emma Christiaen, Nicolas Wyseure, David K Zach, Lars Buytaert, Annemie Jacobs, Ian Buysschaert, Sander Trenson, Raf Van Hoeyweghen, René Tavernier

**Affiliations:** Department of Cardiology, AZ Sint-Jan Brugge, Ruddershove 10, 8000 Bruges, Belgium; Department of Cardiology, AZ Delta, Roeselare, Belgium; Department of Cardiology, Medical University Graz, Graz, Austria; Department of Cardiology, Cliniques Universitaires St. Luc, Brussels, Belgium; Centrum voor Hart- en Vaatziekten, Universitair Ziekenhuis Brussel, Vrije Universiteit Brussel, Jette, Belgium; Department of Cardiology, AZ Sint-Jan Brugge, Ruddershove 10, 8000 Bruges, Belgium; Interuniversity Centre for Biostatistics and Statistical Bioinformatics, KU Leuven, Leuven, Belgium; Interuniversity Centre for Biostatistics and Statistical Bioinformatics, UHasselt, Hasselt, Belgium; Interuniversity Centre for Biostatistics and Statistical Bioinformatics, KU Leuven, Leuven, Belgium; Interuniversity Centre for Biostatistics and Statistical Bioinformatics, UHasselt, Hasselt, Belgium; Department of Cardiology, AZ Sint-Jan Brugge, Ruddershove 10, 8000 Bruges, Belgium; Department of Cardiology, AZ Sint-Jan Brugge, Ruddershove 10, 8000 Bruges, Belgium; Department of Cardiology, AZ Delta, Roeselare, Belgium; Department of Cardiology, Medical University Graz, Graz, Austria; Centrum voor Hart- en Vaatziekten, Universitair Ziekenhuis Brussel, Vrije Universiteit Brussel, Jette, Belgium; Department of Cardiology, AZ Sint-Jan Brugge, Ruddershove 10, 8000 Bruges, Belgium; Department of Cardiology, AZ Sint-Jan Brugge, Ruddershove 10, 8000 Bruges, Belgium; Department of Cardiology, AZ Sint-Jan Brugge, Ruddershove 10, 8000 Bruges, Belgium; Department of Geriatrics, AZ Sint-Jan Brugge, Bruges, Belgium; Department of Cardiology, AZ Sint-Jan Brugge, Ruddershove 10, 8000 Bruges, Belgium

**Keywords:** Octogenarian, Tafamidis, Transthyretin, Cardiac amyloidosis, Cardiomyopathy, Mortality

## Abstract

**Background and Aims:**

In real-world, wild-type transthyretin cardiomyopathy is increasingly diagnosed in patients ≥ 80 years old (octogenarians), although being underrepresented in randomized clinical trials. Specific data on natural course and outcome under tafamidis treatment in octogenarians are therefore scarce. The impact of tafamidis treatment on mortality in real-world wild-type transthyretin cardiomyopathy octogenarians was studied.

**Methods:**

An international, multicentre cohort study of 710 consecutive wild-type transthyretin cardiomyopathy patients with mean follow-up of 2.2 ± 1.8 years for all-cause mortality endpoint was performed.

**Results:**

The cohort consisted of 58.5% (415/710) octogenarians (85 ± 4 years, 74.2% male). Before tafamidis availability, natural course in octogenarians (148/257) vs. non-octogenarians (109/257) was poor, with 16% 1-year and 71% 5-year mortality vs. 8% and 47%, respectively (*P* < .001). Since tafamidis availability, 70.1% (253/361) octogenarians were initiated on tafamidis vs. 83.7% (231/276) non-octogenarians (*P* < .001). Tafamidis discontinuation was similar (octogenarians 10.3% and non-octogenarians 7.4%; *P* = .260). Overall tafamidis treated vs. untreated octogenarians had better unadjusted survival (*P* < .001), with 5% 1-year and 24% 3-year mortality. Tafamidis treatment associated with lower mortality after propensity score matching on baseline variables, including age, National Amyloidosis Centre stage, and New York Heart Association class in on average 394 subjects [hazard ratio (HR) = 0.53, 95% confidence interval (CI) 0.34–0.84, *P* = .007], also in octogenarians (HR = 0.57, 95% CI 0.33–1.01, *P* = .053). Neither age at diagnosis (*P* = .217) nor at treatment initiation (*P* = .154) interacted with tafamidis mortality benefit. Octogenarians had poorer survival despite tafamidis, when initiated at ≥90 years (HR = 3.3, 95% CI 1.10–9.53, *P* = .033) and National Amyloidosis Centre Stage ≥3 (HR = 2.4, 95% CI 0.87–6.46, *P* = .090).

**Conclusions:**

Real-world tafamidis treatment improves survival without age affecting treatment efficacy, although mortality remains considerable in octogenarians.

## Structured Graphical Abstract

**Structured Graphical Abstract:**
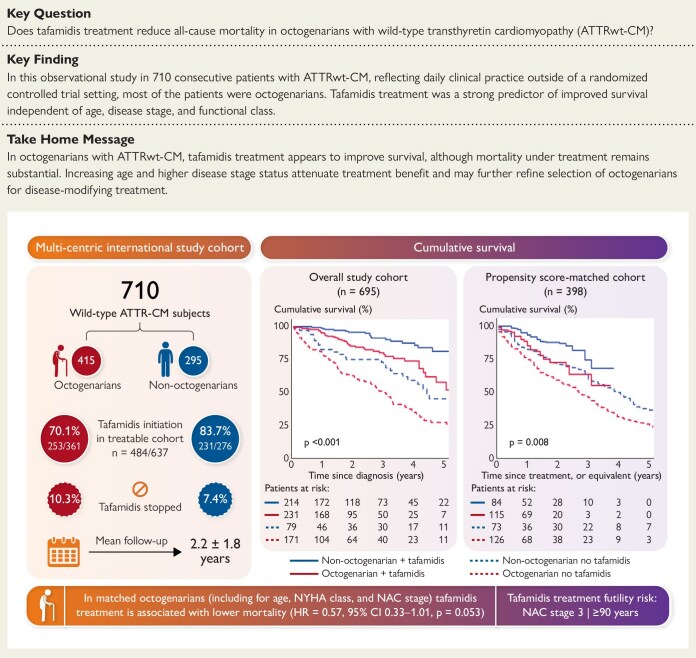
Treatable cohort: since tafamidis availability for wild-type transthyretin cardiomyopathy indication. ATTRwt-CM, wild-type transthyretin cardiomyopathy; CI, confidence interval; HR, hazard ratio; NAC, National Amyloidosis Centre prognostic stage; NYHA, New York Heart Association.

**See the editorial comment for this article ‘Wild-type transthyretin cardiac amyloidosis: the need for a comprehensive geriatric assessment beyond age’, by M. Fontana *et al*., https://doi.org10.1093/eurheartj/ehae491.**

## Introduction

Wild-type transthyretin cardiomyopathy (ATTRwt-CM) is a systemic cardiac disorder that results from extracellular myocardial deposition of insoluble amyloid fibrils, formed by dissociation of transthyretin transporter protein, not related to transthyretin gene mutation.^1^ It is a progressive and age-related condition, diagnosed on average at the age of 74 years old with poor prognosis if left untreated, as evidenced by a median survival of 3.6 years, although depending on disease stage.^1–3^ Exact disease prevalence is unknown but steeply increases with age, rendering octogenarians particularly prone to this disease. Autopsy studies have indeed shown cardiac amyloid deposition in up to 25% of patients ≥ 85 years old and attributed this deposition as the cause of death in 4.5% of octogenarians.^4,5^ Increased awareness and non-invasive diagnostic methods explain the tremendous surge in diagnosed ATTRwt-CM patients seen worldwide.^1^ This in concert with ageing and growing life expectancy in developed countries translates into a future increased likelihood of particularly octogenarians being diagnosed with ATTRwt-CM, as often noted in daily real-world clinical practice.^6,7^ The natural disease course in octogenarians specifically has not been detailed so far.

Tafamidis, an oral tetrameric stabilizer, has emerged as a valuable treatment option for ATTRwt-CM heart failure patients. The ATTR-ACT randomized controlled trial randomized 441 ATTR-CM patients (76.0% wild-type and 24.0% mutant-type) to tafamidis meglumine 80 mg, 20 mg, or placebo once daily in a 2:1:2 ratio.^8^ The study demonstrated a halt in disease progression, as evidenced by less functional decline, reduced quality of life impairment, lower cardiovascular heart failure hospitalizations, and, importantly, an impressive all-cause mortality reduction of 30% in favour of tafamidis.^8^ Tafamidis free acid 61 mg (the bioequivalent of tafamidis meglumine 80 mg) is currently approved by the European Medicines Agency for ATTRwt-CM indication.^9^ More recently, however, a *post hoc* analysis on 353 patients that were randomized between tafamidis meglumine 80 mg and placebo in the ATTR-ACT trial and ongoing Long-Term Extension (LTE) study revealed that only 44 (12.5%) octogenarians with ATTRwt-CM were treated with tafamidis meglumine 80 mg.^9^ Although tafamidis exerted beneficial effects in octogenarians by slowing down functional and quality of life decline with less cardiac biomarker increase, it failed to show significant effect on all-cause mortality in octogenarians [hazard ratio (HR) = 0.68, 95% confidence interval (CI) 0.40–1.15, *P* = .1526]. The effect of tafamidis treatment on mortality in real-world ATTRwt-CM octogenarians, treated outside the context of a randomized trial, is currently unknown. This question is pertinent and clinically relevant, however, as treatment decisions in octogenarians rely on treatment efficacy, coinciding with the complexity of limited life span, polypharmacy, comorbidities, frailty, functional capacity, patient desire, and socio-familial context.^3^

The aim of our study in a contemporary large real-world ATTRwt-CM population, focussing on octogenarians, was therefore three-fold: (i) to describe the natural disease course, (ii) to evaluate the effect of tafamidis treatment on all-cause mortality, and (iii) to explore determinants of tafamidis outcome response. We hypothesized that a significant treatment effect of tafamidis on survival in octogenarian ATTRwt-CM subjects can be detected.

## Methods

### Patient population

Consecutive patients that presented with ATTRwt-CM between October 2009 and December 2023 at five (supra)regional heart centres (four Belgian and one Austrian) were entered in local cardiac amyloidosis registries and included in the current analysis. Patients with mutation detected on serum transthyretin gene analysis were excluded. Wild-type transthyretin cardiomyopathy was diagnosed, conform current recommendations: (i) transthyretin amyloid detection on endomyocardial biopsy, (ii) extracardiac transthyretin positive biopsy with suspect non-invasive cardiac imaging, and/or (iii) Perugini ≥grade 2 myocardial tracer uptake on 99-technetium bone scintigraphy with single-photon emission tomography, without monoclonality, based on serum and urine electrophoresis and normal serum free light chain ratio.^10^

### Baseline patient evaluation

Baseline patient demographics, cardiovascular risk factors, medication use, cardiovascular history, tenosynovial history, and symptoms were collected from the electronic patient files. Cardiac biomarkers, including N-terminal pro-B-type natriuretic peptide (NT-proBNP) and high-sensitive troponin, and estimated glomerular filtration rate (eGFR), were inventoried from serum laboratory testing. Prognostic National Amyloidosis Centre (NAC) ATTR-CM disease stage was determined, applying previously validated NT-proBNP and eGFR cut-offs (Stages I–III).^11^ All patients underwent baseline transthoracic echocardiography. Chamber quantification and function, in addition to valve function, were assessed, as recommended.^12–14^

### Tafamidis treatment

Treatment of ATTRwt-CM with tafamidis free acid 61 mg once daily was initiated at the discretion of the treating physician. Reasons for not initiating or discontinuation of this treatment were collected from a thorough electronic patient chart review. In Belgium, tafamidis was available for ATTRwt-CM treatment indication since August 2020 via a medical need programme for patients with New York Heart Association (NYHA) functional Classes I–III and was subsequently reimbursed since October 2021 for heart failure ATTRwt-CM patients with NYHA functional Classes I and II (with a continued medical need programme for NYHA III functional class subjects already initiated previously). In Austria, tafamidis was reimbursed by insurance companies between July 2018 and February 2021 based on individual approval decisions. Additionally, it was available between December 2018 and March 2020 via a compassionate use programme, where patient selection was at the discretion of the treating physician. Since March 2021, tafamidis is regularly reimbursed for heart failure ATTRwt-CM patients with NYHA functional Class I–III.

The ‘overall study cohort’ included all patients from ATTRwt-CM diagnosis onwards to death or last clinical follow-up, irrespective of tafamidis use and availability. A ‘natural course cohort’ was formed including subjects diagnosed before and censored at the date of tafamidis availability for ATTRwt-CM indication in the respective countries. Finally, the ‘treatable cohort’ consisted of patients alive at the time of tafamidis availability, rendering them potential tafamidis treatment candidates.

### Study endpoint

Patients were followed at the outpatient heart failure clinic on average every 6–12 months. The study endpoint was all-cause mortality, available for all study subjects as the electronic patient files are automatically coupled with the governmental National Death Registers. Patients that discontinued tafamidis treatment were included for endpoint analysis as intention to treat.

The vast majority of patients provided written informed consent. Due to the retrospective nature of the study, need for written informed consent in the remainder was waived by the respective ethical committees. The study complies with standards of the Good Clinical Practice and the Declaration of Helsinki.

### Statistics

Continuous characteristics were summarized using their mean and standard deviation or median and interquartile range. Categorical data were summarized using observed frequencies and percentages. Continuous data were compared using a *t*-test or Wilcoxon rank-sum test, whichever is applicable. Categorical variables were compared using a *χ*^2^ test or Fisher’s exact test, as appropriate. Missing values for all variables were accounted for using multiple imputations with the fully conditional specification method. A total of 20 imputations were performed. The imputation model omitted variables with more than 30% missing values. The variables used in the model are listed in Supplementary data online, *Table S1*.

The study population was stratified by age <80 (non-octogenarians) and ≥80 years (octogenarians) at ATTRwt-CM diagnosis. For the whole study cohort and natural course cohort, Kaplan–Meier curves were provided for time from ATTRwt-CM diagnosis to death. Confidence intervals were calculated using the log(-log)-transformation. In the natural course cohort, predictors of mortality in octogenarians were analysed using a Cox proportional hazard model univariably. Reasons for treatment non-initiation or discontinuation are described descriptively.

We attempted to balance the tafamidis treated/untreated groups by means of propensity scores. The probability of being treated with tafamidis was predicted over time using a Fine and Grey model for competing risk data. Death was considered as a competing risk for being treated with tafamidis. All variables used in the multiple imputation model were also used in this model except the death status and the cumulative baseline hazard for death. Harrell’s *C*-statistic for goodness of fit was calculated using the method described by Wolbers *et al*.^15^

A plot of propensity scores over time for all patients is given in Supplementary data online, *Figure S1*. Patients treated with tafamidis were matched with untreated patients who were still alive at the time that the treatment started for the treated patient and had a similar propensity score. A greedy matching algorithm on the logit of the propensity score, using a calliper of 0.2 times the standard deviation (i.e. 0.2 × 1.05 = 0.21), was applied, and each patient could be selected only once. This matching procedure was repeated separately over the 20 different imputations, resulting in 20 different cohorts of matched patients. Twenty-two patients who were lost to follow-up immediately after being treated were considered as ‘untreated’ during the matching process. An equivalent time of treatment was calculated for non-treated patients by adding the time from diagnosis to treatment of the matched patients to the age of diagnosis of the non-treated patient. In Supplementary data online, *Figure S2*, a graphical representation for an example patient is shown.

All-cause mortality from time of treatment or equivalent was compared between matched treated and untreated patients within each imputed data set using standard methodology for the analysis of survival data (Kaplan–Meier and log-rank tests). These results were combined into a final result using standard combining rules.

Cox regression models for all-cause mortality from time of treatment or equivalent were performed with the tafamidis treatment, age at diagnosis, and age at treatment or equivalent as covariates. The interactions between both ages and tafamidis treatment were verified. The proportional hazard assumptions were tested using the supremum test. For each model, HRs with associated 95% CIs are reported. In addition, we investigated the treatment effect of tafamidis within the octogenarians. Restricted to octogenarians receiving tafamidis treatment, the effect of age, NAC stage, and NYHA class at start of treatment was investigated using Kaplan–Meier curves and a Cox regression model.

All analyses have been performed using SAS software, version 9.4 of the SAS System for Windows. All tests were two sided at a significance level of 5%. Due to the study's exploratory nature, no adjustment was made for multiple testing.

## Results

### Baseline characteristics

**Figure 1:**
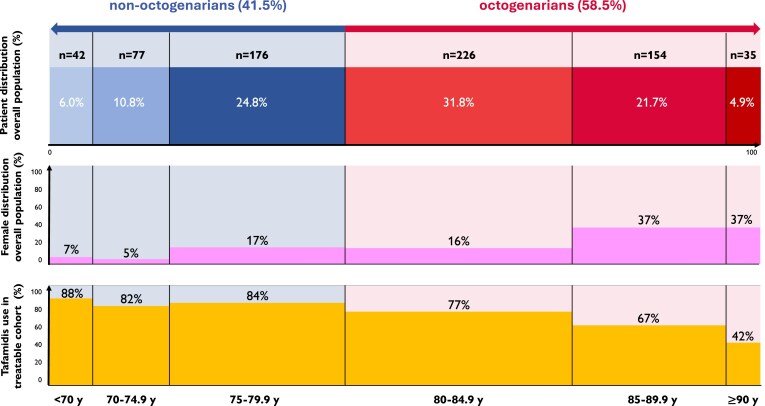
Distribution per age category of patients and female sex in overall study cohort (*n* = 710) and tafamidis treatment in treatable cohort (*n* = 637). y, years old

The overall study cohort comprised 710 patients, including 58.5% (415) octogenarians with age range of 80.0–97.6 years old and 41.5% (295) non-octogenarians, ranging 45.4–79.9 years old (*Figure 1*; *Structured Graphical Abstract*). Patients originated from the cohorts of Roeselare, Bruges (EC number 2674), Graz (EC number 30-286 ex 17/18), Brussels UCL, and Brussels VUB in 39.2% (278, from April 2010 to September 2023), 34.2% (243, from January 2014 to December 2023), 14.4% (102, from July 2015 to March 2023), 6.6% (47, one from October 2009 and rest from March 2016 to August 2023), and 5.6% (40, from September 2018 to October 2023), respectively. Overall, 20.3% (144) of the patients were female with female sex prevalence progressively increasing with age (*P* < .001). In total, 25.8% (107) of octogenarians were female, compared with 12.5% (37) of non-octogenarians (*P* < .001) (*Table 1*). Despite similar degree of structural and systolic functional abnormalities on echocardiography and cardiac biomarker elevation, octogenarians were more symptomatic and presented with more valvular heart disease and higher NAC stage. In concert with comorbidities such as more atrial fibrillation and diastolic and renal dysfunction, this relates to higher history of prior heart failure hospitalization. Bilateral carpal tunnel syndrome, however, was less prevalent in octogenarians. Mean age at ATTRwt-CM diagnosis did not differ over the various years of recruitment (*P* = .468), with the vast majority of 667/710 (94%) included patients being diagnosed since 2016.

**Table 1:** Overall study cohort baseline characteristics, stratified by octogenarian age-category

	Overall cohort *n* = 710	Non-octogenarians *n* = 295	Octogenarians *n* = 415	*P*-value
**Demographics**				
Age, years	[710] 81 ± 7	[295] 74 ± 5	[415] 85 ± 4	**<**.**001**
Male	566/710 (79.7%)	258/295 (87.5%)	308/415 (74.2%)	**<**.**001**
Height, cm	[674] 171 ± 9	[281] 173 ± 9	[393] 169 ± 9	**<**.**001**
Weight, kg	[675] 77 ± 14	[281] 81 ± 15	[394] 73 ± 13	**<**.**001**
Sinus rhythm	328/595 (55.1%)	165/260 (63.5%)	163/335 (48.7%)	.**001**
SBP, mmHg	[587] 135 ± 22	[257] 135 ± 21	[330] 135 ± 23	.955
DBP, mmHg	[587] 76 ± 14	[257] 77 ± 12	[330] 75 ± 15	.**008**
**Cardiovascular risk**				
Smoking history	218/643 (33.9%)	111/275 (40.4%)	107/368 (29.1%)	.**001**
Hyperlipidaemia	403/707 (57.0%)	167/295 (56.6%)	236/412 (57.3%)	.911
Systolic arterial hypertension	448 708 (63.3%)	186/295 (63.1%)	262/413 (63.4%)	.962
Diabetes mellitus	135/708 (19.1%)	58/295 (19.7%)	77/413 (18.6%)	.762
**Medication**				
Beta-blocker	385/710 (54.2%)	155/295 (52.5%)	230/415 (55.4%)	.491
ACE-I/AIIRB/ARNI	302/710 (42.5%)	129/295 (43.7%)	173/415 (41.7%)	.397
Loop diuretic	422/708 (59.6%)	144/295 (48.8%)	278/413 (67.3%)	**<**.**001**
Aldosterone antagonist	259/710 (36.5%)	107/295 (36.3%)	152/415 (36.6%)	.877
Oral anticoagulant	405/709 (57.1%)	155/295 (52.5%)	250/414 (60.4%)	.**044**
Oral antiaggregant	193/709 (27.2%)	80/295 (27.1%)	113/414 (27.3%)	.922
**Cardiovascular history**				
Heart failure hospitalization	212/704 (30.1%)	64/294 (21.8%)	148/410 (36.1%)	**<**.**001**
Coronary artery disease	231/707 (32.7%)	79/295 (26.8%)	152/412 (36.9%)	.**004**
Atrial fibrillation	395/708 (55.8%)	144/294 (49.0%)	251/414 (60.6%)	.**008**
Pacemaker	125/710 (17.6%)	47/295 (15.9%)	78/414 (18.8%)	.300
CRT	57/705 (8.1%)	29/293 (9.9%)	28/412 (6.8%)	.143
ICD	20/705 (2.8%)	13/293 (4.4%)	7/412 (1.7%)	.**032**
Cardiac surgery	94/708 (13.3%)	34/294 (11.6%)	60/414 (14.5%)	.246
Stroke or TIA	102/708 (14.4%)	32/295 (10.8%)	70/413 (16.9%)	.**036**
**Tenosynovial history**				
Bilateral carpal tunnel	251/708 (35.5%)	129/294 (43.9%)	122/414 (29.5%)	**<**.**001**
Lumbar spinal stenosis	212/707 (30.0%)	86/294 (29.3%)	126/413 (30.5%)	.808
**Laboratory serum**				
NT-proBNP, pg/mL	[516] 2284 (913–4909)	[216] 1544 (590–3376)	[300] 2947 (1211–5944)	.103
Hs-troponin I, ng/L	[67] 42 (20–72)	[22] 37 (16–55)	[45] 43 (22–75)	.409
Hs-troponin T, ng/L	[499] 81 ± 418	[233] 52 ± 49	[276] 104 ± 560	.166
eGFR, mL/min	[511] 57 ± 17	[221] 63 ± 17	[290] 52 ± 16	**<**.**001**
**Symptoms and disease stage**				
NYHA class				.**004**
I	170/682 (24.9%)	89/283 (31.4%)	81/399 (20.3%)	
II	323/682 (47.4%)	129/283 (45.6%)	194/399 (48.6%)	
III	174/682 (25.5%)	60/283 (21.2%)	114/399 (28.6%)	
IV	15/682 (2.2%)	5/283 (1.8%)	10/399 (2.5%)	
NAC stage				**<**.**001**
I	223/428 (52.1%)	126/187 (67.4%)	97/241 (40.2%)	
II	134/428 (31.5%)	44/187 (23.5%)	91/241 (37.8%)	
III	70/428 (16.4%)	17/187 (9.1%)	53/241 (22.0%)	
**Echocardiography**				
IVS thickness, mm	[664] 16 ± 4	[277] 16 ± 4	[387] 16 ± 4	.231
PW thickness, mm	[625] 15 ± 5	[260] 16 ± 6	[365] 15 ± 5	.230
LV EDD diameter, mm	[624] 45 ± 8	[262] 45 ± 9	[362] 45 ± 8	.737
LV ESD, mm	[461] 32 ± 7	[196] 32 ± 7	[265] 32 ± 8	.900
LV ejection fraction, %	[665] 54 ± 11	[277] 54 ± 11	[388] 54 ± 12	.901
E/E′	[550] 15 ± 8	[232] 14 ± 8	[318] 16 ± 9	.**009**
sPAP, mmHg	[571] 34 ± 11	[228] 33 ± 11	[343] 35 ± 11	.**004**
TAPSE, mm	[409] 19 ± 6	[175] 16 ± 9	[234] 16 ± 6	.542
TR ≥ moderate	42/688 (6.1%)	6/284 (2.1%)	36/404 (8.9%)	**<**.**001**
MR ≥ moderate	26/688 (3.8%)	5/284 (1.8%)	21/404 (5.2%)	.**019**
AS ≥ moderate	48/690 (7.0%)	6/284 (2.1%)	42/406 (10.3%)	**<**.**001**

Data are represented by [*n*] means ± standard deviation, [*n*] median (Q1–Q3), or numbers and percentages.

Bold indicates statistically significant *P*-value.

ACE-I, angiotensin-converting enzyme inhibitor; AIIRB, angiotensin-2 receptor blocker; ARNI, angiotensin receptor–neprilysin inhibitor; AS, aortic valve stenosis; BSA, body surface area; CRT, cardiac resynchronization therapy device; DBP, diastolic blood pressure; Hs, high-sensitive; EDD, end-diastolic diameter; eGFR, estimated glomerular filtration rate; ESD, end-systolic diameter; ICD, implantable cardioverter defibrillator device; IVS, interventricular wall thickness; LV, left ventricular; NAC, National Amyloidosis Centre; MR, mitral valve regurgitation; NT-proBNP, N-terminal pro-B-type natriuretic peptide; NYHA, New York Heart Association; PW, posterior wall thickness; SBP, systolic blood pressure; sPAP, systolic arterial pulmonary artery pressure; TAPSE, tricuspid annular plane systolic excursion; TIA, transient ischaemic attack; TR, tricuspid valve regurgitation.

### Natural course

**Figure 2:**
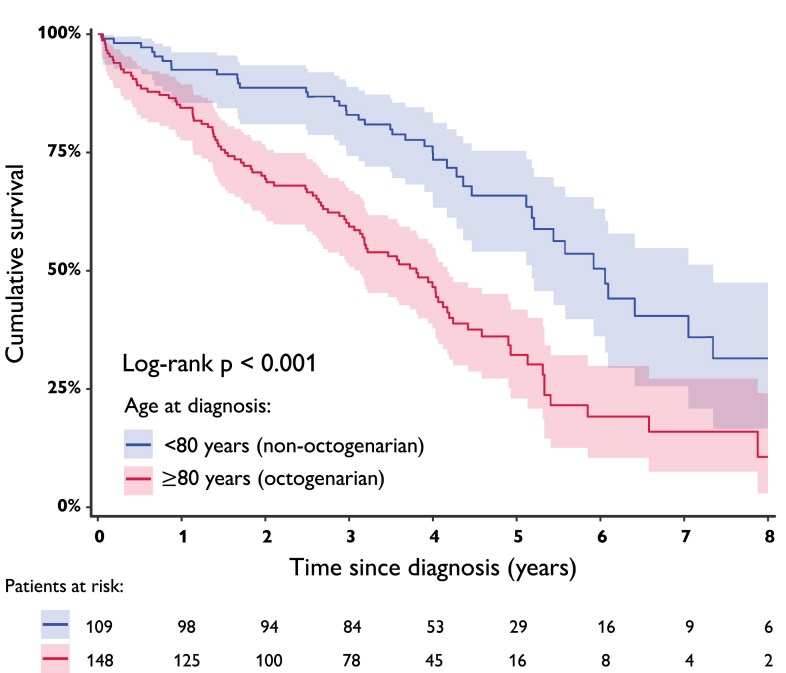
Natural course cohort cumulative survival, stratified by octogenarians and non-octogenarians

The ‘natural course cohort’ was formed by 257 ATTRwt-CM patients [79.8% male, median age 81.0 (77.5–85.2) years old], including 57.6% (148) octogenarians. Overall, median estimated survival was 4.2 years, with 27.2% (70) deaths after a mean follow-up of 1.7 ± 1.6 years. Untreated octogenarians had a poor prognosis, which was worse than untreated non-octogenarians with a median survival of 3.8 vs. 6.1 years (*P* < .001). One- and 5-year mortality was 16% and 71% in octogenarians, compared with 8% and 47% in non-octogenarians, respectively (*Figure 2*). Predictors of mortality in octogenarians included NAC stage (HR = 1.91, 95% CI 1.12–3.24, *P* = .017), in addition to a tendency of both age (HR = 1.08, 95% CI 0.99–1.17, *P* = .059) and NYHA functional class (HR = 1.38, 95% CI 0.99–1.92, *P* = .058). Median survival for NAC Stages I–III in octogenarians was ∼4 years (3.82, 95% CI 3.30–4.34), 3 years (2.97, 95% CI 2.20–3.73), and 2 years (2.01, 95% CI 0.55–3.48), respectively.

### Tafamidis initiation and discontinuation

The ‘treatable study cohort’ (potential tafamidis treatment candidates) comprised 637 ATTRwt-CM patients (79.8% male, mean age 81 ± 7 years), including 361 (74.0% male, mean age 85 ± 4 years) octogenarians (*Structured Graphical Abstract*).

Tafamidis treatment was not initiated in 29.9% (108/361) octogenarians, more than the 16.3% (45/276) in non-octogenarians (*P* < .001). An age-related progressive decline in tafamidis treatment initiation was noted from the age onset of 80 years old (*P* < .001) (*Figure 1*). Reasons for not initiating tafamidis treatment in real world differed between octogenarians and non-octogenarians (*P* = .008) (*Table 2*). In octogenarians, treatment was mostly not initiated due to comorbidities or frailty, advanced age with presumed treatment futility, or advanced disease stage/heart failure. Octogenarians without vs. with tafamidis treatment initiation were older (86 ± 5 vs. 85 ± 3 years, *P* < .001) and more frequently female patients (36.1% vs. 21.7%, *P* = .004) with higher heart failure hospitalization history (41.5% vs. 29.8%, *P* = .031) and had more frequent significant valvular heart disease (all *P* < .050). Nevertheless, similar heart failure medication use, NT-proBNP, and eGFR levels as well as myocardial wall thickness and systolic function were noted (all *P* > .050). Only a tendency towards higher NYHA functional class (*P* = .058) and prognostic NAC stage was seen (*P* = .087).

**Table 2:** Reasons for tafamidis treatment non-initiation in treatable patient cohort

Principal reason	Overall *n* = 153	Non-octogenarians *n* = 45	Octogenarians *n* = 108
Comorbidities and/or frailty	33 (21.6%)	10 (22.2%)	23 (21.3%)
Advanced age, presumed futility	20 (13.1%)	0 (0.0%)	20 (18.5%)
No MNP initiation, died before reimbursement	21 (13.7%)	5 (11.1%)	16 (14.8%)
Advanced disease stage and/or heart failure	21 (13.7%)	6 (13.3%)	15 (13.9%)
Undefined reason	18 (11.8%)	3 (6.7%)	15 (13.9%)
Early disease stage and/or asymptomatic	10 (6.5%)	3 (6.7%)	7 (6.5%)
Lost follow-up and/or treated in other centre	9 (5.9%)	5 (11.1%)	4 (3.7%)
Insurance reimbursement decline	5 (3.3%)	3 (6.7%)	2 (1.9%)
Initiation scheduled next outpatient visit	4 (2.6%)	3 (6.7%)	1 (0.9%)
Incompatible with other studies	4 (2.6%)	3 (6.7%)	1 (0.9%)
Patient preference	8 (5.2%)	4 (8.9%)	4 (3.7%)

MNP, medical need programme.

Tafamidis treatment discontinuation was noted in 8.9% (43/484) of patients after a mean of 552 ± 315 treatment days, without a significant difference between 10.3% (26/253) octogenarians and 7.4% (17/231) non-octogenarians (*P* = .260). Progressive disease with advanced heart failure was the most common discontinuation reason in 39.5% (17/43) of overall subjects (*Table 3*). These patients transitioned to palliative care: 29.4% (5/17) of patients died after a mean follow-up of 0.5 ± 0.6 years, translating into a 1-year mortality of 43%. Presumed treatment side-effects accounted for 16.3% (7/43) of cases.

**Table 3:** Reasons for tafamidis discontinuation in treated patients

Principal reason	Overall *n* = 43	Non-octogenarians *n* = 17	Octogenarians *n* = 26
Progressive disease, advanced heart failure	17 (39.5%)	7 (41.2%)	10 (38.5%)
Undefined reason	8 (18.6%)	2 (11.8%)	6 (23.1%)
Comorbidities, cognitive decline and/or frailty	5 (11.6%)	2 (11.8%)	3 (11.5%)
Advanced age, presumed futility	3 (7.0%)	0 (0.0%)	3 (11.5%)
Side-effects	7 (16.3%)	4 (23.5%)	3 (11.5%)
Patient preference	2 (4.7%)	2 (11.8%)	0 (0%)
Early disease stage, no heart failure	1 (2.3%)	0 (0.0%)	1 (3.8%)

### Tafamidis outcome in overall cohort

In total, 68.2% (484/710) subjects of the overall study cohort received tafamidis treatment throughout their disease course. Tafamidis treated vs. untreated patients were at time of ATTRwt-CM diagnosis on average 3 years younger and more likely to be male, had more tenosynovial red flag history and less significant valvular disease, and were less symptomatic with lower NAC disease stage (11% vs. 29% NAC Stage III, respectively), translating into lower heart failure hospitalization history (23% vs. 45%, respectively) (see Supplementary data online, *Table S2*). Myocardial wall thickness and left ventricular ejection fraction were similar.

Octogenarians (253) vs. non-octogenarians (231) were less initiated on tafamidis treatment (61.1% vs. 78.0%, *P* < .001, respectively). At time of tafamidis therapy initiation, mean age was 85 ± 3 years in octogenarians and 75 ± 6 years in non-octogenarians (*P* < .001). Octogenarians tended to have smaller lag time between ATTRwt-CM diagnosis and tafamidis initiation [70 (19–293) days vs. 92 (30–371) days, *P* = .066]. At start of tafamidis therapy, octogenarians vs. non-octogenarians had higher NAC stage (Stage ≥2 in 55.8% vs. 29.7%, respectively, *P* < .001) and NYHA functional class (Stage ≥III in 26.5% vs. 18.1%, respectively, *P* < .001).

**Figure 3:**
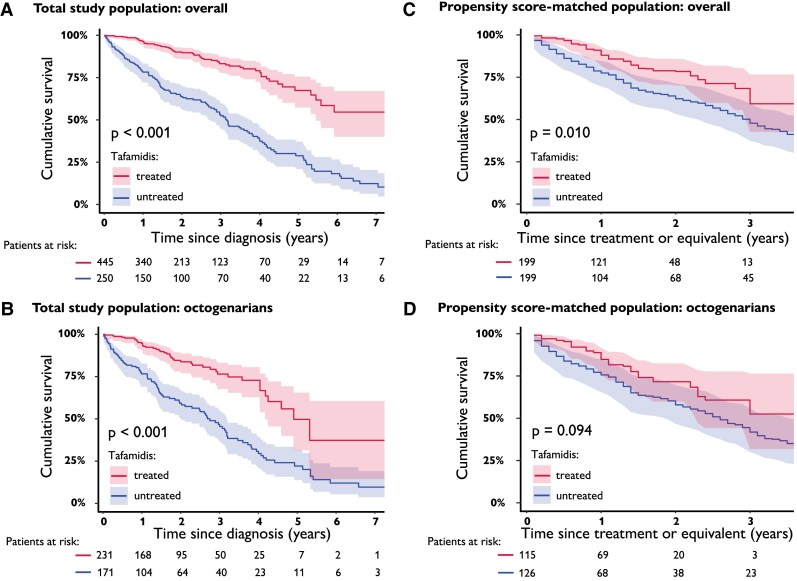
Cumulative survival in tafamidis treated vs. untreated patients. Total patient population. (*A*) Overall cohort. (*B*) Octogenarian cohort. Propensity score-matched population, with patients at risk from the first imputation. (*C*) Overall cohort. (*D*) Octogenarian cohort

Survival analysis was performed on 695 patients, after excluding 15 subjects due to tafamidis initiation date inconsistency. After a mean follow-up of 2.18 ± 1.81 years, a total of 27.9% (194; 129 without and 65 with treatment) patients died. Mortality was lower in tafamidis treated patients both overall (*P* < .001) and in octogenarians (*P* < .001) (*Structured Graphical Abstract*; *Figure 3A* and *B*). One- and 3-year mortality since diagnosis in treated vs. untreated patients was 4% and 17% vs. 22% and 47%, respectively, overall, and 5% and 24% vs. 23% and 54%, respectively, in octogenarians. Tafamidis treatment compared with non-treatment related to a (nearly) two-fold median survival increase in both octogenarians (2.7–4.9 years) and non-octogenarians (4.2–10.3 years).

### Tafamidis outcome in propensity score-matched cohort

A propensity score-matched population of on average 394 patients (range 386–402) was formed. Harrel’s *C*-statistic for the propensity model was 0.63. This population included for instance in the first imputation 61% (241/398) octogenarians, of which 47.7% (115/241) in the tafamidis treated and 52.3% (126/241) in the untreated group. The treated and untreated groups were adequately matched for age, baseline demographics, cardiovascular risk, heart failure medication use, cardiovascular and tenosynovial history, renal function, serum cardiac biomarkers, and echocardiographic structure and function, in addition to functional NYHA class and NAC disease stage (all *P* = NS; *Table 4*).

**Table 4:** Baseline characteristics stratified by tafamidis treatment category for propensity score-matched population

	Untreated *n* = 199	Treated *n* = 199	*P*-value
**Demographics**			
Age, years	81 ± 7	81 ± 6	.523
Male	147 (73.9%)	157 (78.9%)	.238
Height, cm	170 ± 9	169 ± 9	.507
Weight, kg	75 ± 14	76 ± 14	.638
Sinus rhythm	105 (52.8%)	96 (48.2%)	.761
SBP, mmHg	133 ± 26	134 ± 21	.751
DBP, mmHg	75 ± 16	75 ± 12	.976
**Cardiovascular risk**			
Smoking history	68 (34.1%)	66 (33.2%)	.976
Hyperlipidaemia	112 (56.3%)	113 (56.8%)	.919
Systolic arterial hypertension	122 (61.3%)	124 (62.3%)	.837
Diabetes mellitus	40 (20.1%)	37 (18.6%)	.703
**Medication**			
Beta-blocker	114 (57.3%)	111 (55.8%)	.762
ACE-I/AIIRB/ARNI	79 (39.7%)	77 (38.7%)	.837
Loop diuretic	120 (60.3%)	127 (63.8%)	.470
Aldosterone antagonist	66 (33.2%)	71 (35.7%)	.598
Oral anticoagulant	115 (57.8%)	123 (61.8%)	.413
Oral antiaggregant	52 (26.1%)	54 (27.1%)	.821
**Cardiovascular history**			
Heart failure hospitalization	69 (34.7%)	61 (30.7%)	.393
Coronary artery disease	68 (34.2%)	78 (39.2%)	.298
Atrial fibrillation	109 (54.8%)	119 (59.8%)	.311
Pacemaker	27 (13.6%)	39 (19.6%)	.106
CRT	13 (6.5%)	22 (11.1%)	.111
ICD	5 (2.5%)	8 (4.0%)	.398
Cardiac surgery	30 (15.1%)	32 (16.1%)	.782
Stroke or TIA	29 (14.6%)	31 (15.6%)	.779
**Tenosynovial history**			
Bilateral carpal tunnel	68 (34.2%)	74 (37.2%)	.530
Lumbar spinal stenosis	57 (28.6%)	60 (30.2%)	.741
**Laboratory serum**			
NT-proBNP, pg/mL	3389 (1322–7038)	3407 (1420–6346)	.857
Hs-troponin T, ng/L	82 (36–216)	63 (53–113)	.079
eGFR, mL/min	56 ± 18	55 ± 16	.466
**Symptoms and disease stage**			
NYHA class			.780
I	52 (26.1%)	53 (26.6%)	
II	86 (43.2%)	86 (43.2%)	
III	60 (30.2%)	57 (28.6%)	
IV	1 (0.5%)	3 (1.5%)	
NAC stage			.222
I	76 (37.8%)	73 (36.3%)	
II	80 (39.8%)	94 (46.8%)	
III	42 (21.4%)	32 (16.1%)	
**Echocardiography**			
IVS thickness, mm	16 ± 4	17 ± 4	.165
PW thickness, mm	15 ± 5	15 ± 5	.536
LV EDD diameter, mm	45 ± 9	45 ± 8	.590
LV ejection fraction, %	54 ± 12	55 ± 12	.216
E/E′	16 ± 10	16 ± 8	.918
sPAP, mmHg	35 ± 11	34 ± 12	.627
TAPSE, mm	19 ± 6	19 ± 6	.843
TR ≥ moderate	11 (5.5%)	12 (6.0%)	.830
MR ≥ moderate	7 (3.5%)	10 (5.0%)	.457
AS ≥ moderate	15 (7.5%)	14 (7.0%)	.847

Results corresponding to imputation number 1 only. Data are represented by means ± standard deviation, median (Q1–Q3), or numbers and percentages. All variables were included in the propensity scoring model. For abbreviations, see legend *Table 1*.

Patients with vs. without tafamidis treatment had overall lower mortality since tafamidis treatment (or equivalent) (*P* = .007) (*Figure 3C*). One- and 3-year mortality rates in tafamidis treated vs. untreated patients were 12% and 40% vs. 23% and 52%, respectively. In octogenarians, 1- and 3-year mortality rates were 15% vs. 46% and 25% vs. 57%, respectively (*Structured Graphical Abstract*; *Figure 3D*). During follow-up since treatment (or equivalent), a total of 31.7% (136/199; 93 without and 43 with treatment) patients died in the first imputed data set. Lower mortality since tafamidis treatment (or equivalent) was noted both in the overall propensity score-matched population (HR = 0.53, 95% CI 0.34–0.84, *P* = .007) and the octogenarian subgroup (HR = 0.57 95% CI 0.33–1.01, *P* = .053).

**Figure 4:**
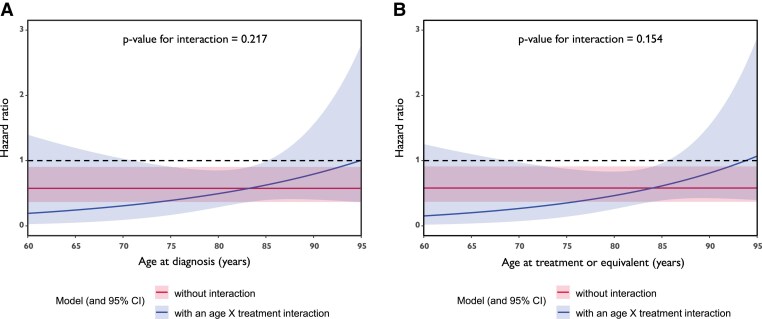
Age interaction with tafamidis treatment efficacy (mortality). (*A*) Interaction with age at diagnosis. (*B*) Interaction with age at start treatment (or equivalent). CI, confidence interval

Importantly, Cox proportional hazard regression for mortality since tafamidis treatment (or equivalent) showed tafamidis relates to better survival, irrespective of age at ATTRwt-CM diagnosis (*P* = .019) or age at treatment initiation (*P* = .021) or both (*P* = .020) (*Table 5*). Although age at diagnosis and age at treatment initiation relate to mortality, both do not attenuate tafamidis treatment efficacy, illustrated by the absence of interaction effect for both variables throughout the entire age spectrum (*P* = NS) (*Figure 4*).

**Table 5:** Cox proportional hazard regression for all-cause mortality since tafamidis treatment or equivalent for propensity score-matched population

Predictor	Hazard ratio (95% CI)	*P*-value
Model 1: Baseline model		
Tafamidis treatment	0.52 (0.33–0.84)	.**007**
Model 2: Age at diagnosis^a^		
Tafamidis treatment	0.56 (0.35–0.91)	.**019**
Age at diagnosis (years)	1.07 (1.04–1.11)	**<**.**001**
Model 3: Age at start treatment^b^		
Tafamidis treatment	0.56 (0.35–0.92)	.**021**
Age at start treatment or equivalent (years)	1.08 (1.04–1.12)	**<**.**001**
Model 4: Age at diagnosis and at start treatment		
Tafamidis treatment	0.56 (0.34–0.91)	.**020**
Age at diagnosis (years)	0.84 (0.68–1.05)	.116
Age at start treatment or equivalent (years)	1.29 (1.03–1.60)	.**025**

Bold indicates statistically significant *P*-value.

CI, confidence interval.

^a^No significant interaction between age at diagnosis and tafamidis treatment (*P* = .217).

^b^No significant interaction between age at start treatment and tafamidis treatment (*P* = .154).

### Octogenarian mortality under tafamidis treatment

**Figure 5:**
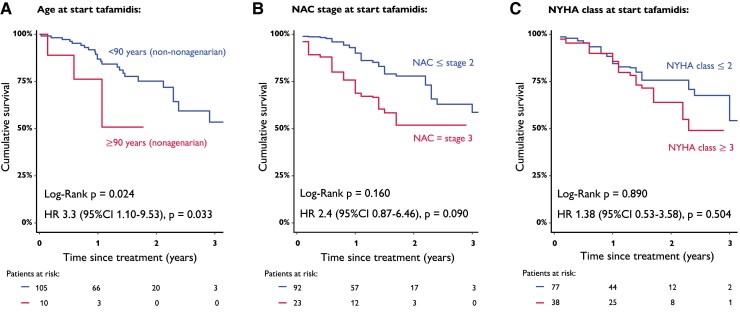
Cumulative survival in tafamidis treated octogenarians, since treatment. (*A*) Stratified by nonagenarian and non-nonagenarian. (*B*) Stratified by National Amyloidosis Centre prognostic stage category. (*C*) Stratified by New York Heart Association functional class category. CI, confidence interval; HR, hazard ratio; NAC, National Amyloidosis Centre; NYHA, New York Heart Association

We evaluated determinants of outcome since treatment, restricted to octogenarians receiving tafamidis treatment. In this population, higher age at tafamidis therapy initiation showed increased mortality risk (HR = 3.3, 95% CI 1.10–9.53, *P* = .033). Particularly nonagenarians (≥90 years old) compared with non-nonagenarians had higher 1-year mortality of 38% and 15%, respectively (*Figure 5A*). In addition, NAC disease Stage 3 at start of tafamidis treatment showed a tendency towards higher mortality in octogenarians (HR = 2.4, 95% CI .87–6.46, *P* = .090). One-year mortality of NAC Stage 3 vs. NAC Stage ≤2 octogenarian tafamidis treated patients was 30% vs. 10%, respectively (*Figure 5B*). Higher NYHA functional class, however, was not significantly associated to increased mortality risk under treatment (HR = 1.38, 95% CI 0.53–3.58, *P* = .504). One-year mortality in NYHA ≥III vs. ≤II patients was 13% and 16%, respectively (*Figure 5C*). This analysis should be regarded as exploratory, at the verge of a relatively limited number of patients and follow-up time.

## Discussion

The main study findings on real-world ATTRwt-CM patients are as follows: (i) octogenarians represent the vast majority of patients diagnosed in daily clinical practice, (ii) octogenarians have poor natural course, (iii) tafamidis treatment relates to improved survival, including in octogenarians, and (iv) age does not interact with tafamidis efficacy.

Although the exact ATTRwt-CM disease prevalence is unknown, the strong age-related incidence implies octogenarians are a specific population of interest.^9^ Indeed, our results indicate octogenarians represent up to 58.5% of encountered ATTRwt-CM patients in daily life. The prevalence of amyloid deposition on myocardial histology in octogenarian autopsy series is as high as 20%–25% but of variable intensity, translating into a 5%–10% prevalence of ATTRwt-CM clinical phenotype.^1,5,16,17^ Another autopsy study on 490 unselected octogenarians demonstrated that 4.5% (22) patients died from (visually and histologically) overt cardiac amyloidosis with ante-mortem heart failure symptoms.^4^ This would translate into an estimation of over 1.2 million deaths attributed to cardiac amyloidosis in Europe, based on 27.1 million octogenarians living in Europe in 2022 (source: Eurostat online). Given the age-related prevalence and increasing life expectancy, one could even expect a significant rise of future octogenarians presenting with ATTRwt-CM.^3,7^ Nevertheless, the recent randomized controlled ATTR-ACT trial and LTE study failed to represent this age prevalence on inclusion, with only 24.9% (88/353) of octogenarians being studied and therefore may not be generalizable to the general ATTRwt-CM population, as indicated before.^9,18^ The recent ATTRibute-CM trial, however, randomized 51.1% (312/611) patients ≥ 78 years old to acoramidis 800 mg twice daily or placebo in a 2:1 ratio, another oral tetrameric stabilizer, but overall failed to demonstrate all-cause mortality benefit as a secondary outcome, potentially related to earlier disease stage inclusion.^19^ Treatment outcome in octogenarians with ATTRwt-CM should be the scope of future studies and randomized trials. Another striking finding and reported before is the age-related female prevalence in ATTRwt-CM, with 25.8% of octogenarians in our study cohort being female, implying ATTRwt-CM screening should not be restricted to elderly males only.^20^ Females, however, only represented 9.8% of inclusion in both the ATTR-ACT and ATTRiBUTE-CM trial.^8,19^ Finally, octogenarians typically present with more heart failure symptoms and later disease stage, in line with previous publications.^9^

Although the natural course in ATTRwt-CM has been extensively documented and prognostic disease stages using biomarkers are well defined, data on octogenarians in particular have not been reported to date.^2,11,21^ We demonstrated poor overall survival in untreated octogenarians with high 1- and 5-year mortality rates of 16% and 71%, respectively, and a median survival of 3.8 years. Previous studies documented a median survival of 2.2–5.6 years in an overall untreated population of ATTRwt-CM subjects with 5-year mortality up to 64%.^1,2,21,22^ A median survival of 4.8 years was noted in another large cohort of both wild-type and mutant-type ATTR-CM patients.^11^ In addition, we showed that NAC disease stages related to all-cause mortality in octogenarians, with median survival of 4, 3, and 2 years in NAC Stage I–III octogenarians, respectively, compared with ∼6, 4, and 2 years in an overall population of 869 both wild-type and mutant-type ATTR-CM subjects.^11^

Treatment decision-making at the discretion of the treating physician in real-world elderly patients significantly differs from the context of a randomized controlled trial applying strict in- and exclusion criteria. Treatment initiation and discontinuation, specifically in octogenarians, need to be well balanced and result from a more challenging integration of treatment efficacy and complex biological factors (comorbidities, polypharmacy, frailty, mobility, and cognition) that co-determine outcome, at the verge of debatable cost-effectiveness of (tafamidis) treatment in patients with intrinsically limited lifespan.^3,23,24^ Nevertheless, average life expectancy in developed countries for overall White octogenarian females and males is 9.1 and 7.0 years, respectively.^6^ Median lifespan in nonagenarians, however, is 4–5 years.^23^ Although tafamidis treatment is projected to result in a significant 1.29 additional quality-adjusted life years, a recent US cost-effectiveness study indicated a 92.6% price reduction would be needed to assure cost-effectiveness of tafamidis.^24^ Treatment access and affordability remain a major challenge for ATTRwt-CM management, particularly as the disorder affects often elderly patients and a combination of various (novel and highly expensive) ATTRwt-CM disease-modifying therapies may well prove to be most effective in the future, further increasing the healthcare expenditure burden.

In the current report, we inventoried for the first time, to the best of our knowledge, reasons for not initiating tafamidis in (elderly) ATTRwt-CM patients as well as reasons for treatment discontinuation. Comorbidities (including frailty), advanced age, and advanced heart failure accounted for over half of reasons not to start tafamidis treatment in octogenarians. Interestingly, 6.5% of octogenarians were not initiated due to early disease stage or asymptomatic status; whether asymptomatic early-stage patients benefit from treatment remains to be elucidated. Treatment discontinuation was noted in only 8.9%, without difference between octogenarians and non-octogenarians and less compared with 21.2% in the treated arm of the ATTR-ACT trial.^8^ Comorbidities and progressive disease account for approximately half of the reasons to stop treatment, with only 16.3% being attributed to presumed side-effects, confirming general tolerability and drug safety in the real world.

Although octogenarians represent the majority of ATTRwt-CM patients evaluated in the real world, the recent ATTR-ACT trial and LTE study included a limited number of 44 octogenarian patients only on active tafamidis meglumine 80 mg (bioequivalent to currently approved tafamidis free acid 61 mg) treatment and excluded nonagenarians.^19,22^ In the *post hoc* analysis of these studies, no mortality benefit of tafamidis treatment in octogenarians was demonstrated (*P* = .1526), probably related to the study being underpowered for this not prespecified analysis in a limited number of patients.^19^ Therefore, the mortality benefit of tafamidis treatment in octogenarians remains unproven and complicates contemporary decision-making in the real world. Our observational study, however, included 253 real-world octogenarian patients under tafamidis treatment and suggested overall mortality benefit, including in octogenarians (HR = 0.57, 95% CI 0.33–1.01, *P* = .053), when adequately matched for a variety of baseline variables, including independent predictors such as age, baseline NAC disease stage, and NYHA functional class. Importantly, we additionally showed that age at ATTRwt-CM diagnosis nor at tafamidis initiation did affect treatment survival benefit, indicating expected survival benefit of tafamidis treatment is similar for younger vs. elderly patients.

### Clinical implications

In line with our study results, one should consider tafamidis treatment to improve survival in ATTRwt-CM patients, including octogenarians, who represent ∼60% of real-world ATTRwt-CM patients. Nevertheless, mortality under treatment in matched octogenarians remains substantial with 15% 1-year and 46% 3-year mortality since treatment (comparable with octogenarians in the ATTR-ACT trial).^9^ In addition, we showed in an exploratory analysis that very advanced age (nonagenarians) and potentially advanced disease stage (NAC Stage 3) may attenuate mortality benefit despite tafamidis treatment in octogenarians. This implies careful individualized decision-making for initiating disease-modifying treatment within this age category. In summary, these parameters may therefore prove to be helpful to rationalize the initiation of treatment in selected octogenarians that are likely to benefit most of treatment, preferentially within a multidisciplinary team, including a geriatrician, although further confirmatory study is warranted.

### Strengths and limitations

Current study represents the largest real-world study population on tafamidis treated all-cause mortality outcome in octogenarians with ATTRwt-CM, a factor six-fold compared with the limited number of treated octogenarians evaluated in the ATTR-ACT and LTE study.^9^ In addition, it is the first study evaluating ATTRwt-CM natural disease course in octogenarians specifically and detailing reasons to initiate or discontinue tafamidis treatment in a large real-world population of ATTRwt-CM patients.

Some limitations should be acknowledged. First, our analysis was limited to the all-cause mortality endpoint and did not evaluate tafamidis treatment effect on functional capacity, quality of life neither NT-proBNP. The beneficial effect of tafamidis in octogenarians on the latter variables was, however, recently demonstrated in the ATTR-ACT trial and LTE study.^9^ Second, due to the observational nature of our study in real-world patients, tafamidis treatment selection bias is immanent, potentially favouring tafamidis treatment initiation in more vital octogenarians with less comorbidity and better intrinsic life expectancy. We showed, however, a survival benefit of tafamidis treatment in octogenarians, after propensity score matching for a wide spectrum of baseline variables including age, disease stage, heart failure symptoms and medication use, and well-known and strong prognostic factors. Third, NYHA functional class heterogeneity throughout inclusion may have resulted from the heterogeneous criteria for tafamidis eligibility during the inclusion period. Fourth, the use of sodium-glucose transport 2 inhibitors could potentially be a mortality confounder; however, these data were unavailable for analysis but merit future study in the context of ATTRwt-CM. Finally, frailty was recently shown to be a predictor of survival, after correction for disease stage, NYHA class, and tafamidis use (but not age), present in up to 39% of ATTRwt-CM patients.^25^ Data on frailty metrics were not available, however, and therefore could not be accounted for as a potential modulator of survival under tafamidis treatment.

## Conclusion

In real-world clinical practice, octogenarians represent the vast majority of ATTRwt-CM patients. Our data suggest that tafamidis treatment relates to lower mortality, matched for of age, NAC disease stage and NYHA functional class, and without interaction between age and treatment efficacy. Therefore, tafamidis treatment may be considered in ATTRwt-CM octogenarians to improve survival. Nevertheless, mortality under treatment remains considerable and its predictors need further study to rationalize the selection of optimal octogenarian treatment candidates. Octogenarian ATTRwt-CM patients should urgently become the scope of future clinical trial design and inclusion to obtain objective results on treatment efficacy and safety that help guide multidisciplinary and complex, individualized decision-making. In addition, further research should determine optimal octogenarian candidates for disease-modifying treatments, in terms of benefit vs. futility due to very early asymptomatic or too advanced stage.

## Supplementary data

Supplementary data are available at *European Heart Journal* online.

## Supplementary Material

ehae923_Supplementary_Data

## Declarations

### Data Availability

Data can be shared upon reasonable request and after approval of all participating centres.

### Pre-registered Clinical Trial Number

None supplied.
