# Supplementary material for: Tafamidis in octogenarians with wild-type transthyretin cardiac amyloidosis: an international cohort study
Source: Eur Heart J. 2025 Feb 26;46(11):1057–70. doi: 10.1093/eurheartj/ehae923 (PMC11905754; doi:10.1093/eurheartj/ehae923)
Supplement: ehae923_Supplementary_Data [file ehae923_supplementary_data.pdf]

Supplementary Figure 1. Propensity scores.

Panel A: propensity scores by Tafamidis treatment.

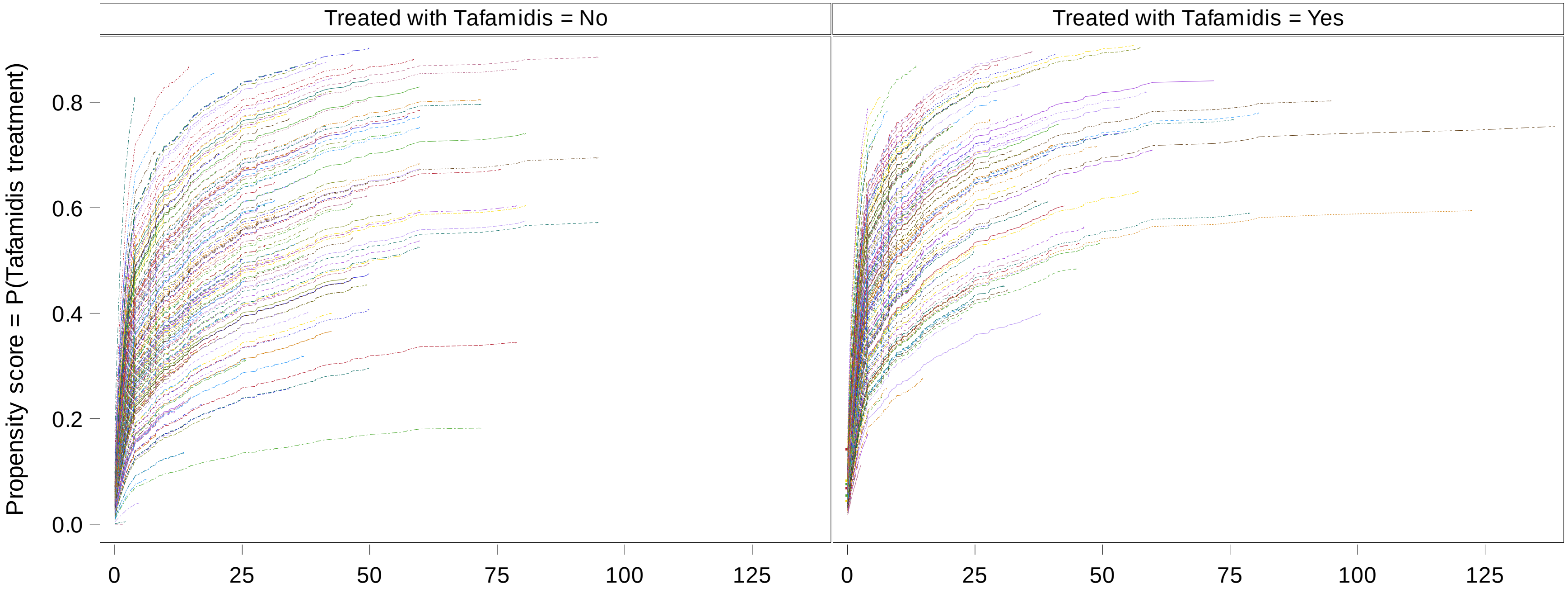

The propensity scores are the probabilities over time of being treated with Tafamidis at a specific time, as estimated by the propensity score model. For untreated patients, the curves stop at the time of censoring or patient death. For treated patients, the curves stop at the time of treatment. Treated patients are matched to untreated patients who have similar propensity scores at the same time as the treatment started and who are still alive and in the study at the time. Controls (no treatment) can only be selected once.

Panel B: Example plot showing the propensity score-matching for the first imputation (out of 20). No suitable control could be found for n = 246 patients who were treated with Tafamidis. The figure illustrates close matching for most of the matched patients.

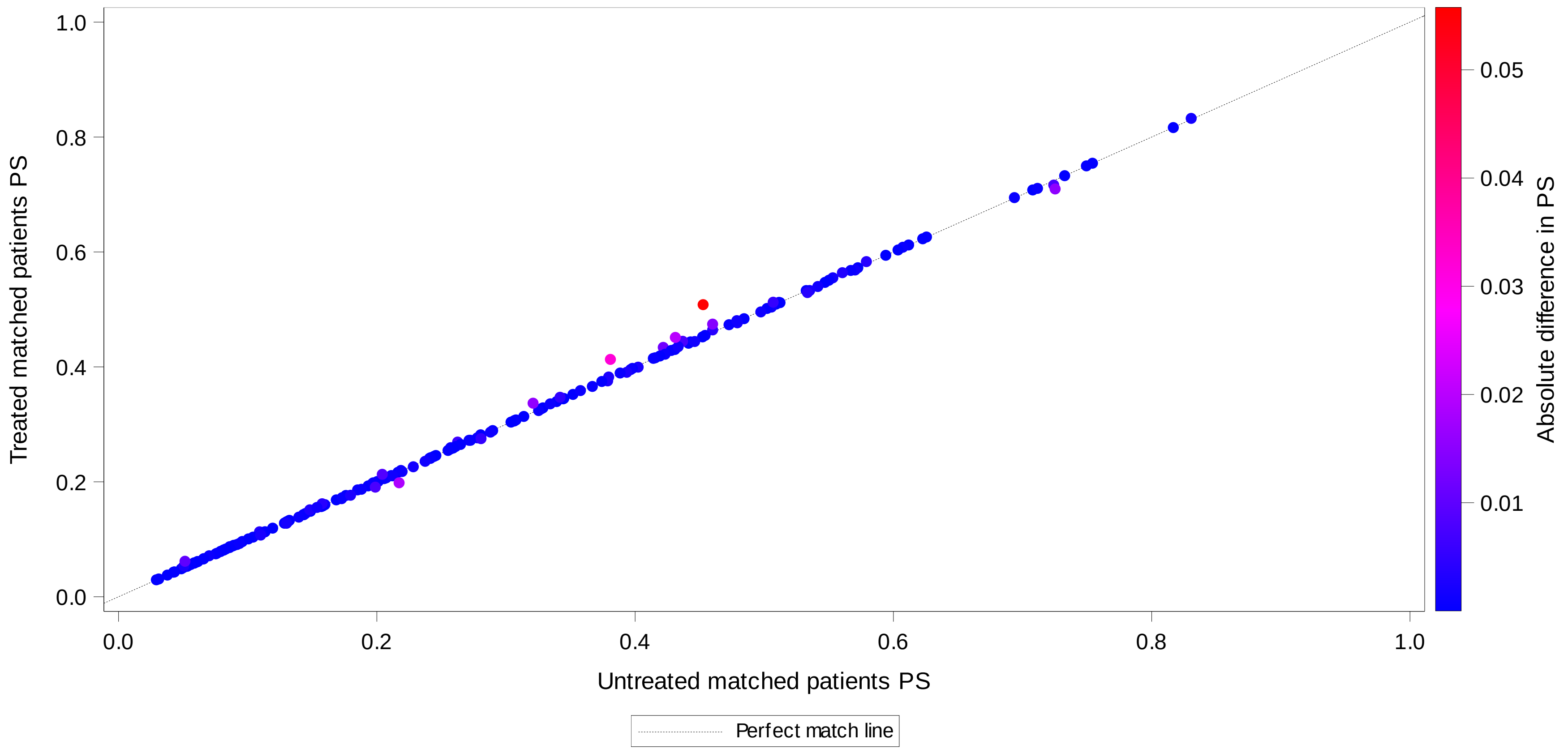

Supplementary Figure 2. Illustration of the equivalent treatment time for an untreated matched patient.

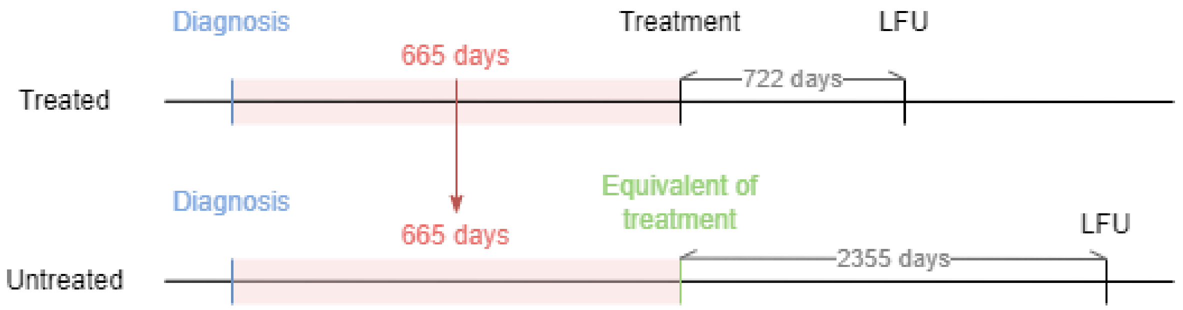

As an example, a patient treated with Tafamidis on day 665 (approximately 1.8 years) after being diagnosed with ATTR had a propensity score (estimated probability being treated with Tafamidis according to the propensity model) of 0.827. This patient was matched to a patient who never received Tafamidis treatment, and who had a propensity score of 0.828 on day 665 after being diagnosed with ATTR and was still alive at that time (equivalent of treatment). A control patient could only be matched once, i.e. after matching this patient was removed from the pool. The matching was done using a greedy matching algorithm on the logit of the propensity score, using a calliper of 0.2 times the standard deviation (i.e. 0.2 x 1.05 = 0.21). A fairly wide calliper was chosen to enable as many decent matches as possible. No suitable match was found for 270 treated patients.

Supplementary Table 1. Variables included in the multiple imputation model.

Variables
**Demographics**
Patient origin
Age at ATTR diagnosis, years
Male gender
Height, cm
Weight, kg
Sinus rhythm
SBP, mmHg
DBP, mmHg
**Cardiovascular risk**
Smoking history
Hyperlipidaemia
Systolic arterial hypertension
Diabetes mellitus
**Medication**
Beta-blocker
ACE-I/AIIRB/ARNI
Loop diuretic
Aldosterone antagonist
Oral anticoagulant
Oral antiaggregant
**Cardiovascular history**
Heart failure hospitalization
Coronary artery disease
Atrial fibrillation
Pacemaker
CRT
ICD
Cardiac surgery
Stroke or TIA
**Tenosynovial history**
Bilateral carpal tunnel
Lumbar spinal stenosis
**Laboratory serum**
NT-proBNP, pg/mL
Hs-Troponin T, ng/L
eGFR, mL/min
**Symptoms and disease stage**
NYHA class
NAC stage*
**Echocardiography**

IVS thickness, mm
PW thickness, mm
LV EDD diameter, mm
LV ejection fraction, %
E/E’
sPAP, mmHg
TAPSE, mm
TR ≥moderate
MR ≥moderate
AS ≥ moderate
**Outcome**
Outcome: All-cause death
Cumulative baseline hazard for death

*The NAC (National Amyloidosis Centre) stages were not imputed but were recomputed within each imputation using the following rules: NAC stage 1 if NT-proBNP≤ 3.000 pg/mL and eGFR ≥45 ml/min; NAC stage 3 if NT-proBNP >3.000 pg/mL and eGFR <45 ml/min; NAC stage 2 otherwise. ACE-I: angiotensin converting enzyme inhibitor; AIIRB: angiotensin-2 receptor blocker; AMI: acute myocardial infraction, ARNI: angiotensin receptor neprilysin inhibitor; AS: aortic valve stenosis; BSA: body surface area; ATTR: Transthyretin cardiac amyloidosis; CABG: coronary artery bypass grafting; CAD: coronary artery disease; CRT: cardiac resynchronization therapy device; DBP: diastolic blood pressure; Hs: high-sensitive; EDD: end-diastolic diameter; eGFR: estimated glomerular filtration rate; ESD: end-systolic diameter; ICD: implantable cardioverter defibrillator device; IVS: interventricular wall thickness; LV: left ventricular; NAC: National Amyloidosis Centre prognostic stage; MR: mitral valve regurgitation; NT-proBNP: N-terminal pro-brain natriuretic peptide; NYHA: New York Heart Association dyspnea class; PCI: percutaneous coronary intervention; PW: posterior wall thickness; SBP: systolic blood pressure; sPAP: systolic arterial pulmonary artery pressure; TAPSE: tricuspid annular plane systolic excursion; TIA: transient ischemic attack; TR: tricuspid valve regurgitation.

Supplementary Table 2. Overall study cohort baseline characteristics, stratified by Tafamidis treatment category.

	Overall cohort n=710	Untreated n=226	Treated n=484	p-value
**Demographics**				
Age, years	[710] 81±7	[226] 83±7	[484] 80±7	**<0.001**
Male	566/710 (79.7%)	164/226 (72.3%)	402/484 (83.1%)	**0.001**
Height, cm	[674] 171±9	[199] 169±9	[475] 171±8	**<0.001**
Weight, kg	[675] 77±14	[201] 74±14	[474] 78±14	**<0.001**
Sinus rhythm	328/595 (55.1%)	82/158 (51.9%)	246/437 (56.3%)	0.135
SBP, mmHg	[587] 135±22	[158] 131±26	[429] 136±20	**0.008**
DBP, mmHg	[587] 76±14	[158] 73±15	[429] 77±13	**0.002**
**Cardiovascular risk**				
Smoking history	218/643 (33.9%)	62/201 (30.9%)	156/442 (35.2%)	0.511
Hyperlipidaemia	403/707 (57.0%)	126/2246 (56.3%)	277/483 (57.4%)	0.783
Systolic arterial hypertension	448/708 (63.3%)	132/224 (58.9%)	316/484 (65.3%)	0.103
Diabetes mellitus	135/708 (19.1%)	41/224 (18.3%)	94/484 (19.4%)	0.725
**Medication**				
Beta-blocker	385/710 (54.2%)	128/226 (56.6%)	257/484 (53.1%)	0.378
ACE-I/AIIRB/ARNI	302/710 (42.5%)	91/226 (40.3%)	212/484 (43.8%)	0.375
Loop diuretic	422/708 (59.6%)	150/224 (67.0%)	272/484 (56.2%)	**0.007**
Aldosterone antagonist	259/710 (36.5%)	76/226 (33.6%)	183/484 (37.8%)	0.281
Oral anticoagulant	405/709 (57.1%)	130/226 (57.2%)	275/483 (56.9%)	0.883
Oral antiaggregant	193/709 (27.2%)	65/226 (27.4%)	131/483 (27.1%)	0.931
**Cardiovascular history**				
Heart failure hospitalization	212/704 (30.1%)	99/222 (44.6%)	113/482 (23.4%)	**<0.001**
Coronary artery disease	231/707 (32.7%)	79/224 (35.3%)	152/483 (31.5%)	0.316
Atrial fibrillation	395/708 (55.8%)	131/225 (58.2%)	264/483 (54.7%)	0.374
Pacemaker	125/709 (17.6%)	36/225 (16.0%)	89/484 (18.4%)	0.437
CRT	57/705 (8.1%)	12/226 (5.3%)	45/479 (9.39%)	0.063
ICD	20/705 (2.8%)	6/226 (2.7%)	14/479 (2.9%)	0.842
Cardiac surgery	94/708 (13.3%)	34/225 (15.1%)	60/483 (12.4%)	0.326

Stroke or TIA	102/708 (14.4%)	38/225 (16.9%)	64/483 (13.3%)	0.199
**Tenosynovial history**				
Bilateral carpal tunnel	251/708 (35.5%)	64/225 (28.4%)	187/483 (38.7%)	**0.008**
Lumbar spinal stenosis	212/707 (30.0%)	55/225 (24.4%)	157/482 (32.6%)	**0.028**
**Laboratory serum**				
NT-proBNP, pg/mL	[516] 2284 (916-4872)	[146] 3352 (1322-7131)	[370] 2017 (833-4338)	**<0.001**
Hs-Troponin I, ng/L	[67] 42 (20-72)	[32] 43 (27-101)	[35] 40 (17-54)	0.149
Hs-Troponin T, ng/L	[499] 48 (29-81)	[147] 66 (35-109)	[352] 44 (27-71)	**<0.001**
eGFR, mL/min	[511] 57±17	[149] 54±19	[362] 58±16	**0.015**
**Symptoms and disease stage**				
NYHA class				**<0.001**
I	170/682 (24.9%)	47/210 (22.4%)	123/472 (26.1%)	
II	323/682 (47.4%)	80/210 (38.1%)	243/472 (51.5%)	
III	174/682 (25.5%)	73/210 (34.8%)	101/472 (21.4%)	
IV	15/682 (2.2%)	10/210 (4.8%)	5/472 (1.1%)	
NAC stage				**<0.001**
I	223/427 (52.2%)	49/125 (39.2%)	174/302 (57.6%)	
II	134/427 (31.4%)	40/125 (32.0%)	94/302 (31.1%)	
III	70/427 (16.4%)	36/125 (28.8%)	34/302 (11.3%)	
**Echocardiography**				
IVS thickness, mm	[664] 16±4	[213] 16±4	[451] 16±4	0.825
PW thickness, mm	[625] 15±5	[203] 15±5	[422] 15±5	0.556
LV EDD diameter, mm	[624] 45±8	[198] 45±8	[426] 45±8	0.757
LV ESD, mm	[461] 32±7	[164] 32±8	[297] 32±7	0.764
LV ejection fraction, %	[665] 54±11	[215] 53±12	[450] 55±11	0.167
E/E'	[550] 15±8	[180] 17±10	[370] 15±7	**0.008**
sPAP, mmHg	[571] 34±11	[186] 36±12	[385] 33±11	**0.002**
TAPSE, mm	[409] 19±6	[141] 18±5	[268] 19±6	0.080
TR ≥moderate	42/688 (6.1%)	28/221 (12.7%)	14/467 (3.0%)	**<0.001**
MR ≥moderate	26/688 (3.8%)	17/221 (7.7%)	9/467 (1.9%)	**<0.001**
AS ≥ moderate	48/690 (7.0%)	26/222 (11.7%)	22/468 (4.7%)	**<0.001**

ACE-I: angiotensin converting enzyme inhibitor; AIIRB: angiotensin-2 receptor blocker; ARNI: angiotensin receptor neprilysin inhibitor; AS: aortic valve stenosis; BSA: body surface area; CRT: cardiac resynchronization therapy device; DBP: diastolic blood pressure; Hs: high-sensitive; EDD: end-diastolic diameter; eGFR: estimated glomerular filtration rate; ESD: end-systolic diameter; ICD: implantable cardioverter defibrillator device; IVS: interventricular wall thickness; LV: left ventricular; NAC: National Amyloidosis Centre prognostic stage; MR: mitral valve regurgitation; NT-proBNP: N-terminal pro-brain natriuretic peptide; NYHA: New York Heart Association dyspnea class; PW: posterior wall thickness; SBP: systolic blood pressure; sPAP: systolic arterial pulmonary artery pressure; TAPSE: tricuspid annular plane systolic excursion; TIA: transient ischemic attack; TR: tricuspid valve regurgitation. Data are represented by [n] means ± standard deviation, [n] median (Q1 – Q3) or numbers and percentages.
